# Rupture of the Bladder from a Blow

**Published:** 1827-05

**Authors:** 

**Affiliations:** the Middlesex Hospital.


CASE II. *Rupture of the Bladder from a Blow.* Treated by Mr. SHAW, at the MIDDLESEX HOSPITAL.

February 5th, 1827.—Thomas Doneghan, aged twenty-two. He had been drinking gin with his friends yesterday, and, in running a race with one of them, he stumbled, and struck the lower part of the belly against a post. From the violence of the blow, he was thrown backwards, and lay for some minutes insensible. He suffered violent pain during the whole day, and this morning he went to a surgeon, who passed a gum elastic catheter, but no urine flowed. He was bled, and very shortly afterwards came to the hospital. He stands half-doubled, his hands pressed upon the lower part of the belly, with an anxious countenance, and imploring assistance.

He complains of urgent pain across the loins and lower part of the abdomen, which is tense, but no well-defined tumor, like the distended bladder, is apparent. Has made no urine since yesterday at half-past one P.M. A cathether was passed, though not without great difficulty, owing to the strong contraction of the muscles in the perineum: here the instrument was literally held fast. About half a pint of bloody-coloured urine, with coagula, were drawn off; but little or no relief followed. The sensation given to the hand was not at all similar to what is felt when the catheter is in the bladder: it had too wide a range, and seemed to hitch against various irregularities.

Twelve leeches to the perineum, and a dose of Castor-oil and Laudanum.—Fomentations to the lower part of the belly and perineum.

Very shortly after being admitted, he vomited a quantity of greenish fluid. The pain continued unabated.

In the evening, he was bled to deliquium (ten ounces) from a large orifice; twelve leeches were applied to the abdomen. The catheter, with great difficulty, was introduced, and a small quantity of urine was drawn off, but with little coagulum.

6th.—Has passed a restless night, pain being constant; and he has continued vomiting the same greenish fluid. No urine has flowed naturally; neither have the bowels acted, although enemata have been administered. He does not feel any desire to evacuate his urine, and, on the catheter being introduced, none passed.

Infus. Rosæ ℥ jss.; Mag. Sulph. ʒj.; Træ. Opii m. viij. secundis horis.—Twelve leeches to the abdomen.—Ext. Opii gr. jss. h. s.

7th.—Vomiting still continues. Complains of racking pain at the lower part of the belly, which the slightest pressure increases. Pulse is small, quick, and hard; the skin is hot, and the tongue furred and dry. He felt a desire to make water to-day, and the catheter was introduced, when two pints of clear-coloured urine flowed. Some degree of relief ensued, but the belly remained hard and distended. Nearly the same quantity of urine was drawn off in the evening.

Hydr. Sub. gr. ij.; Opii gr. ss. sextis horis.—Empl. Lyttæ abdomini.

Feb. 8th.—In the morning the catheter was introduced, and a pint of urine drawn off. His appearance is altered for the worse; the countenance is sunk, and his breathing is laborious. The vomiting continues, and is now decidedly stercoraceous. He lies with his knees drawn up; the abdomen is tense, and cannot bear the pressure of the bedclothes, and each cough or exertion causes him excruciating agony. The bowels, notwithstanding the administration of various enemata, have not been opened; neither has the bladder been relieved, excepting by the assistance of the catheter, which is passed morning and evening. Pulse is becoming small, and the tongue is remarkably clean. But a few drops of urine flowed by the catheter in the evening.

Calomel and Opium omitted in the evening.

9th.—No urine flowed by the catheter when introduced to-day. Pain of the abdomen less since the blister was applied. Pulse is weak, and the tongue is furring. There is no distinct tumor in the abdomen, it is swollen uniformly.

Vin. rubri ℥ j. secunda q. q. hora.

He sunk rapidly, and died at eleven P.M.—Just before death he passed a motion, the only natural one he had since his admission.

*Post-mortem examination.*—Abdomen distended; omentum dark coloured and matted to the intestines, but no adhesion between it and the peritoneal lining of the abdominal muscles. Intestines dark coloured, matted together, and distended, so that the small are as large as the great intestines usually are: patches of coagulable lymph are effused upon their external coat, and the mucous lining is vascular; but no lesions are apparent, and they are quite empty of feces. There is a large collection of fluid in the pelvis, and, on passing a sound into the urethra, it enters the pelvis through a large rent in the left side of the bladder. The fluid is thick, and has the smell of urine. A portion of intestine, which has fallen down into the pelvis, and lies bathed in the urine, is soft, and appears to be gangrened. The rectum is coated with coagulable lymph, and flakes are floating in the fluid. The bladder is a little thickened, but is very partially contracted. There is no eversion of the inner coat. The whole cavity of the abdomen presents the appearance of active inflammation having taken place.

*Remarks.*—In cases of ruptured bladder (fortunately by no means of frequent occurrence,) the surgeon has to lament his want of power to afford relief, and it is with pain that he watches the progress of a case which must inevitably have a fatal termination.

The distended bladder receives a violent blow, and is burst; the urine is extravasated into the cavity of the pelvis, and causes inflammation of the peritoneum, and death. In a case related by Dr. Cusack, in the second volume of the Dublin Hospital Reports, an opening was made into the abdomen, and a large quantity of perfectly transparent urine was evacuated: the patient was relieved for the time, but, as might be expected, the relief was not permanent, and he died on the eighth day. A case is also related in the Sepulchretum of Bonetus, where the abdomen was punctured, but the patient died.

In the first case, from the great and general shock to the system, the patient died before reaction had perfectly taken place, and before inflammation of the peritoneum had fairly commenced.

In the case of Donneghan, as rapid and as acute an inflammation of the intestines seemed to have been produced by the sudden effusion of the urine, as that caused by strangulation of a portion of gut. The intestinal tube being thus disturbed in its functions, the symptoms closely resembled those of ileus. It seemed extraordinary that, on the third day, a large quantity of water should have been drawn off by the catheter; but this was explained on dissection, for a new bladder or sac had been formed by the matting together of the peritoneal surfaces in the lower part of the abdomen. The catheter passed through the rent in the bladder into this sac, which had prevented the urine from being so generally effused as it had been before adhesion took place.

